# Knowledge, Attitude, and Perception of Medical Students Toward AI‐Based Learning: A Cross‐Sectional Study

**DOI:** 10.1002/puh2.70293

**Published:** 2026-06-06

**Authors:** Danyal Khan, Muhammad Dawood, Ihtiram Ullah, Husna Irfan Thalib, Sadia Yasir Khan, Farasat Maheen, Mable Pereira

**Affiliations:** ^1^ Medicine and Surgery Gomal Medical College Dera Ismail Khan Pakistan; ^2^ General Medicine Practice Program Batterjee Medical College Jeddah Saudi Arabia; ^3^ School of Medicine Lincoln American University Georgetown Guyana

**Keywords:** artificial intelligence, attitude, Khyber Pakhtunkhwa, knowledge, medical education, medical students, perception

## Abstract

**Background:**

Artificial intelligence (AI) is rapidly transforming healthcare delivery and medical education. Despite its growing global presence, little is known about the knowledge, attitudes, and perceptions (KAP) of medical students in Pakistan regarding AI‐based learning. Understanding these factors is essential for guiding curriculum development and preparing future physicians for an AI‐driven health system.

**Objective:**

To assess the KAP of medical students in Khyber Pakhtunkhwa (KP), Pakistan, toward AI‐based learning.

**Methods:**

A cross‐sectional survey was conducted among 392 medical students from multiple medical colleges in KP. A structured, self‐administered questionnaire was used to assess KAP toward AI‐based learning. Data were analyzed using SPSS version 26, employing descriptive statistics and chi‐square tests to examine group differences. Ordinal logistic regression was performed to identify independent predictors, with statistical significance set at *p* < 0.05. Ethical approval was obtained from the Institutional Review Board of Gomal Medical College, and informed consent was obtained from all participants.

**Results:**

Of the 392 participants, most demonstrated moderate knowledge (62.8%) regarding AI‐based learning, whereas only 30.4% achieved high knowledge scores. Significant variation was observed across academic years, with senior students demonstrating greater knowledge than preclinical students (*p* < 0.05). In contrast, attitudes and perceptions were largely positive across all years, with nearly three‐quarters (74.8%) of students expressing favorable views toward AI. Only 0.8% reported negative attitudes. These findings suggest that enthusiasm for AI is high, but comprehensive understanding remains limited.

**Conclusion:**

Medical students in KP exhibit strong enthusiasm and positive perceptions toward AI‐based learning, yet their knowledge remains modest and uneven across training levels. This mismatch highlights the urgent need for structured AI education in undergraduate curricula. Incorporating formal training, workshops, and faculty development programs can bridge this gap and better prepare future physicians to responsibly integrate AI into clinical practice.

## Graphical abstract

Artificial intelligence (AI) is rapidly transforming medical education, yet students’ readiness for AI‐based learning in Pakistan remains unclear. This cross‐sectional study of 392 medical students in Khyber Pakhtunkhwa assessed knowledge, attitudes, and perceptions using a structured questionnaire and statistical analysis (*p* < 0.05). Most students demonstrated moderate knowledge (62.8%), with only 30.4% showing high knowledge, whereas senior students performed significantly better than juniors. Despite this, attitudes were largely positive, with 74.8% expressing favorable perceptions and only 0.8% negative views. Overall, although enthusiasm for AI is high, knowledge remains limited, highlighting the need for structured AI integration into undergraduate medical curricula.

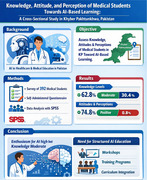

## 1 Introduction

The study of artificial intelligence (AI) is concerned with creating machines that can carry out tasks that ordinarily call for human intelligence [1]. AI has drawn a lot of interest and is being used more and more in a variety of industries around the world, including health. International medical education systems have included AI‐based learning resources, including ChatGPT, IBM Watson, and AI‐powered medical imaging technologies [2]. AI is used in imaging diagnostics, such as in the assessment of skin images or CT scans, among many other applications [3]. By enhancing diagnostic precision, providing individualized learning experiences, and automating a variety of instructional duties, AI is transforming medical education [4]. In recent years, medical practitioners and specialists have become interested in AI due to its wide range of medical applications [5]. In spite of this growth, developing nations, like Pakistan, continue to face difficulties in implementing AI in the healthcare system due to a number of issues, including provider inertia, financial constraints, a lack of qualified health professionals to set up diagnostic protocols on which algorithms are based, fear of physician replacement, social barriers, confidentiality, and medico legal implications [6]. Although some students in Pakistan are hopeful about AI in medical education, many are still unaware of its full potential [7]. The infrastructure for medical education differs in Khyber Pakhtunkhwa (KP), and there is currently little information on how students feel about AI. In order to support decision makers, inform curriculum creation, and close the gap between innovation and real‐world implementation, it will be helpful to investigate their awareness, perspectives, and readiness.

Medical students’ awareness of AI varies around the world. According to a study conducted in Malaysia, only 30% of students were confident in their knowledge of AI [8]. In contrast, research conducted in Europe showed that 60% of students thought AI would have a significant impact on medicine [9]. A study conducted in Jordan revealed that 89% of the students believed in the importance of AI in the medical field, and 71.4% believed in the benefits of teaching AI in the medical career [10]. One study conducted in Arab countries found that 87.1% of students had a low level of knowledge regarding AI, whereas 43.4% of students strongly agreed upon teaching AI in medical curriculum [11]. The integration of AI in medical curricula is still in its infancy in Pakistan, where a recent study found that only 20% of medical students had used ChatGPT for studying, highlighting a gap in AI adoption. According to a different survey that included medical students from all around Pakistan, just 68.8% of them knew the fundamentals of AI, and only 19.4% of them were aware of its uses in medicine [12]. This study demonstrates how medical students, despite having little knowledge and training regarding the use of AI in medicine, acknowledge the importance of AI for healthcare systems and medical curriculum and have a positive image of AI.

Research shows that medical students in KP have varying degrees of expertise on AI. Most medical students (61.7%) have no prior understanding of AI, according to a study done in Peshawar, a significant city in KP [13]. This ignorance emphasizes the necessity of introducing AI principles and applications to medical students through curriculum integration. Unlike prior studies limited to single institutions or national aggregates, this study provides a multi‐institutional analysis across academic levels within KP, offering insights relevant for regional curriculum planning and staged competency development. This study aims to assess how medical students of KPK perceive AI‐based learning, their knowledge about AI applications, and their willingness to integrate it into medical education and practice.

## 2 Materials and Methods

### 2.1 Study Design

This was a cross‐sectional study conducted among undergraduate medical students enrolled in public and private medical colleges across KP. Participants were drawn from all academic years, ranging from first‐ to final‐year MBBS.

### 2.2 Study Population and Sampling

The target population consisted of MBBS students from medical colleges across KP. The study was carried out through the Department of Community Medicine at Gomal Medical College, Dera Ismail Khan. The sample size was determined using the OpenEpi sample size calculator with a 95% confidence interval (CI), a 5% margin of error, and an assumed 50% response distribution. On the basis of an estimated total of 16,000 MBBS students in KP, the minimum required sample size was 376. To account for incomplete or missing responses, the target was set at 400 participants.

Students were eligible for inclusion if they were currently enrolled in public or private medical colleges within KP, belonged to any academic year from first‐ to final‐year MBBS, and provided informed consent to participate in the study. Students from outside KP, those unwilling to participate, individuals who submitted incomplete or duplicate responses, and those who were not undergraduate MBBS students, such as house officers, postgraduate trainees, or consultants, were excluded.

A convenience sampling technique was employed. Students from different institutions and academic years who were accessible and willing to participate were invited to complete the questionnaire. Although efforts were made to ensure proportional representation across years and institutions, logistical limitations precluded strict randomization. This limitation should be borne in mind when interpreting the findings.

### 2.3 Data Collection

Data were collected using a semi‐structured questionnaire developed by adapting items from previously validated tools used in similar studies, with modifications and additional questions incorporated to address the specific objectives of the present research on AI‐based learning among medical students in KP (Supporting Information File 1). To enhance clarity, relevance, and reliability, a pilot test was conducted with 10–15 students. Feedback from this pilot phase informed revisions and refinement of the final questionnaire. Internal consistency of the instrument was assessed using Cronbach's alpha. To check if the instrument was consistent, Cronbach's alpha was used. The questionnaire's overall reliability was considered satisfactory, attaining a total Cronbach's alpha of 0.78. The internal consistency remained robust when disaggregated into subscales: The knowledge domain exhibited an alpha of 0.76, the attitude domain an alpha of 0.81, and the perception domain an alpha of 0.84.

### 2.4 Statistical Analysis

Data were entered into Microsoft Excel and analyzed using SPSS version 26. Descriptive statistics were used to summarize participant characteristics. Categorical variables, such as gender, year of study, and residence status, were presented as frequencies and percentages, whereas continuous variables, including knowledge, attitude, and perception (KAP) scores, were expressed as means and standard deviations. Knowledge responses were scored as “1” for correct and “0” for incorrect answers, with total knowledge scores categorized into low (0–2), moderate (3–4), and high (≥5) levels. Attitude and perception were assessed using six items each on a five‐point Likert scale ranging from 1 (strongly disagree) to 5 (strongly agree). Scores were classified as negative (1–12), neutral (13–15), or positive (16–30). Instead of applying arbitrary cutoff values, the distribution of the collected data was used to determine the thresholds for categorizing KAP scores. The 33rd and 67th percentiles were employed to classify the continuous scores into three categories: low, moderate, and high (or negative, neutral, and positive). This data‐driven approach ensures that the categorization accurately reflects the distribution and relative performance of the study population.

The normality of continuous data was assessed using the Kolmogorov–Smirnov and Shapiro–Wilk tests. As most variables violated the assumption of normality (*p* < 0.05), nonparametric tests were applied. The Kruskal–Wallis test was employed to compare mean ranks of KAP scores across academic years. Ordinal logistic regression analyses were conducted to identify independent predictors of AI KAP. Separate models were built for each outcome variable using academic year, gender, and residential status as independent variables. The proportional odds model was applied, and adjusted odds ratios (aOR) with 95% CI were reported. Variables with a *p* value < 0.05 were considered statistically significant after controlling for potential confounders.

### 2.5 Ethical Considerations

Confidentiality and anonymity were maintained throughout the research process. The research was conducted over a 6‐month period from March 1 to August 31, 2025. No identifiable participant data were transferred outside Pakistan. All datasets were anonymized prior to analysis. The corresponding author contributed to study design, interpretation, and manuscript preparation in compliance with ethical standards.

## 3 Results

### 3.1 Participant Characteristics

A total of 392 medical students participated in the study. The largest proportion of respondents was from Gomal Medical College (*n* = 156, 39.8%), followed by Jinnah Medical College (*n* = 27, 6.9%), Ayub Medical College (*n* = 25, 6.4%), and Khyber Medical College (*n* = 23, 5.9%). Other institutions were represented to a lesser extent, including Khyber Girls Medical College (*n* = 20, 5.1%), Nowshera Medical College (*n* = 23, 5.9%), Rehman Medical College (*n* = 23, 5.9%), Abbottabad International Medical College (*n* = 15, 3.8%), and several others with smaller numbers (Table 1).

**TABLE 1:** Demographic characteristics of study participants (*N* = 392).

Variable	Category	Frequency (*n*)	Percentage
**Gender**	Male	243	62.0
	Female	149	38.0
**Year of study**	First year	62	15.8
	Second year	50	12.8
	Third year	76	19.4
	Fourth year	153	39.0
	Final year	51	13.0
**Residence**	Hostel	269	68.6
	Day scholar	123	31.4
**Institute**	Gomal Medical College	156	39.8
	Jinnah Medical College	27	6.9
	Ayub Medical College	25	6.4
	Khyber Medical College	23	5.9
	Nowshera Medical College	23	5.9
	Rehman Medical College	23	5.9
	Khyber Girls Medical College	20	5.1
	Abbottabad International Med. Col	15	3.8
	Saidu Medical College	11	2.8
	Kabir Medical College	11	2.8
	Bannu Medical College	10	2.6
	Gajju Khan Medical College	7	1.8
	Govt. College of Nursing D.I Khan	7	1.8
	Bacha Khan Medical College	7	1.8
	Northwest Medical College	6	1.5
	Swat Medical College	5	1.3
	Women Medical College	5	1.3
	PMC	8	2.0
	Pak International Medical College	2	0.5
	Sardar Begum Medical College	1	0.3
**Total**		**392**	**100.0**

Among the participants, 243 (62.0%) were male and 149 (38.0%) were female. With respect to academic year, the distribution was as follows: first year (*n* = 62, 15.8%), second year (*n* = 50, 12.8%), third year (*n* = 76, 19.4%), fourth year (*n* = 153, 39.0%), and final year (*n* = 51, 13.0%). Regarding residence status, 269 students (68.6%) resided in hostels, whereas 123 (31.4%) were day scholars.

### 3.2 Knowledge of AI‐Based Learning

The mean overall knowledge score (KNW‐total) was 9.23 (SD = 1.45). On the basis of predefined cutoff values, 27 students (6.9%) had low knowledge, 246 (62.8%) had moderate knowledge, and 119 (30.4%) had high knowledge (Table 2).

**TABLE 2:** Knowledge distribution among years of study.

Year of study	Mean	SD	Range	df	*p* value
First year	4.21	1.46	0–7	4	0.013
Second year	4.80	1.37	1–7	4	0.013
Third year	4.99	1.42	0–7	4	0.013
Fourth year	4.86	1.39	1–7	4	0.013
Final year	4.86	1.64	0–7	4	0.013

When compared across academic years, knowledge scores differed significantly (Kruskal–Wallis *H* = 12.638, df = 4, *p* = 0.013). First‐year students had the lowest mean ranks (153.09), whereas third‐year students had the highest (214.21). Year‐wise mean scores on the 0–7 scale were first year (mean = 4.21, SD = 1.46, range = 0–7), second year (mean = 4.80, SD = 1.37, range = 1–7), third year (mean = 4.99, SD = 1.42, range = 0–7), fourth year (mean = 4.86, SD = 1.39, range = 1–7), and final year (mean = 4.86, SD = 1.64, range = 0–7).

A chi‐square test indicated no significant association between gender and knowledge status (*p* = 0.494).

### 3.3 Perceptions Toward AI‐Based Learning

The distribution of perception scores (per‐total) was strongly positive. A total of 289 students (73.7%) reported positive perceptions, 100 (25.5%) were neutral, and only 3 (0.8%) expressed negative perceptions (Table 3).

**TABLE 3:** Perception scores by academic year.

Year of study	Mean	SD	Range	Positive (%)	Neutral (%)	Negative (%)
First year	24.31	3.16	16–30	73.7	25.5	0.8
Second year	24.04	4.23	6–30			
Third year	24.63	3.49	14–30			
Fourth year	24.69	3.34	15–30			
Final year	24.35	3.78	12–30			

No statistically significant difference in perception was found between academic years (Kruskal–Wallis *H* = 0.910, df = 4, *p* = 0.923). Similarly, no association was observed between institute and perception status (*p* = 0.139).

Year‐wise mean perception scores were first year (24.31, SD = 3.16, range = 16–30), second year (24.04, SD = 4.23, range = 6–30), third year (24.63, SD = 3.49, range = 14–30), fourth year (24.69, SD = 3.34, range = 15–30), and final year (24.35, SD = 3.78, range = 12–30).

### 3.4 Attitudes Toward AI‐Based Learning

Attitudes followed a similar pattern. A total of 291 students (74.2%) reported positive attitudes, 98 (25.0%) were neutral, and only 3 (0.8%) expressed negative attitudes (Table 4).

**TABLE 4:** Attitude scores by academic year.

Year of study	Mean	SD	Range	Positive (%)	Neutral (%)	Negative (%)
First year	20.63	3.50	8–25	74.2	25.0	0.8
Second year	20.68	3.43	12–25			
Third year	20.43	3.72	10–25			
Fourth year	20.76	3.22	10–25			
Final year	20.33	3.54	11–25			

There were no significant differences in attitude scores across study years (Kruskal–Wallis *H* = 0.477, df = 4, *p* = 0.976). Likewise, no association was found between residence status and attitude status (*p* = 0.804).

Year‐wise mean attitude scores were first year (20.63, SD = 3.50, range = 8–25), second year (20.68, SD = 3.43, range = 12–25), third year (20.43, SD = 3.72, range = 10–25), fourth year (20.76, SD = 3.22, range = 10–25), and final year (20.33, SD = 3.54, range = 11–25).

### 3.5 Ordinal Logistic Regression Analysis of Predictors of AI KAP Among Students

An ordinal logistic regression was performed to identify independent predictors of higher AI knowledge scores. The model included academic year, gender, and residential status as independent variables. After accounting for potential confounding variables, academic year remained a significant independent predictor of AI knowledge (*p* = 0.020). The odds of first‐year students demonstrating superior AI knowledge were significantly lower than those of final‐year students (aOR = 0.417, 95% CI: 0.202–0.861, *p* = 0.018). In the adjusted model, gender (*p* = 0.502) and residence status (*p* = 0.941) did not significantly predict knowledge levels.

An identical ordinal logistic regression analysis was used to identify the independent factors that predicted positive attitudes toward AI‐assisted learning. Gender was found to be a significant factor predicting the attitude of the students toward AI (*p* = 0.004). This means that males were much more likely to have positive attitudes toward AI than females (aOR = 1.827, 95% CI: 1.211–2.757, *p* = 0.004). Unlike the case of knowledge, academic year (*p* = 0.846) and place of residence (*p* = 0.977) had no significant influence on attitude.

Finally, an ordinal logistic regression was carried out to determine the independent predictors of positive attitudes toward AI. In line with the attitude theory, the results show that gender is the only independent predictor (*p* = 0.032). The likelihood of having positive attitudes toward AI is almost four times higher for male students than for female students (aOR = 3.972, 95% CI: 1.130–13.967, *p* = 0.032). Neither academic year (*p* = 0.365) nor residence status (*p* = 0.115) proved to be independent predictors of attitudes.

### 3.6 Statistical Considerations

The application of nonparametric methods was justified, as both Kolmogorov–Smirnov and Shapiro–Wilk tests rejected normality for knowledge, perception, and attitude scores across years (*p* < 0.05 in most cases). Consequently, the Kruskal–Wallis test was employed for group comparisons. Knowledge was statistically significantly different across academic years (Kruskal–Wallis *H* = 12.638, df = 4, *p* = 0.013), with a small effect size of 0.032.

There was a significant relationship between gender and positive attitude toward AI‐assisted learning, chi‐square (1, *N* = 392) = 8.24, *p* = 0.004, *V* = 0.14, which is considered small.

## 4 Discussion

This study assessed KAP of AI‐based learning among medical students in KP. Overall, we found a pattern frequently reported in the literature: Students show positive attitudes and perceptions toward AI‐based learning while demonstrating only modest, variable levels of factual knowledge. These findings can be interpreted within the framework of competency‐based medical education and digital health literacy, emphasizing the need to align emerging technological competencies with structured curricular outcomes. Similar national and regional surveys have reported that many students and early career clinicians feel optimistic about AI's role in education and healthcare despite limited formal experience or deep technical understanding [14, 15, 16, 17]. Knowledge appeared to increase with seniority: Students in clinical years scored higher than preclinical students, a finding consistent with other national surveys that associate greater clinical exposure and self‐directed learning with higher familiarity with AI concepts [18]. Internationally, comparable studies from diverse settings (Middle East, Europe, and South Asia) report the same gap, receptive attitudes alongside a fragmented or superficial knowledge base that suggests a global curricular shortfall rather than a region‐specific phenomenon [17, 18, 19].

The predominance of informal learning sources (social and mainstream media and online forums) over structured curricular teaching likely explains this gap. In one multicenter survey, over 80% of students cited media as their primary information source about AI, whereas only a small minority reported formal curricular training. This explains why attitudes are generally positive (influenced by visibility and hype), yet objective knowledge and confidence in using AI clinically are limited [17, 18]. Educational and policy implications are clear. Medical curricula should capitalize on students’ favorable attitudes by introducing structured, scaffolded AI education: foundational concepts (terminology, data basics, and strengths/limitations), applied modules (case‐based use of AI tools, interpretability, and clinical validation), and ethical/legal training (privacy, bias, and accountability). Faculty development, practical workshops, elective projects with data scientists, and partnerships with industry/research groups can accelerate competency building while preserving clinical humanism. These recommendations align with prior calls for curricular integration and competency‐based AI training in medical schools [19, 20, 21].

Strengths of our study include a timely assessment of AI‐related KAP among medical students in KP and the use of a structured questionnaire aligned with prior KAP research. In addition, AI is a rapidly evolving field; students’ knowledge and tools available to them may change quickly, which affects the durability of any cross‐sectional estimate. The World Health Organization and other bodies emphasize both rapid change and the need to embed ethical governance and trustworthiness into AI education, points we must consider when designing curricula [22]. This study has several limitations. First, the use of a cross‐sectional design prevents us from inferring causality between exposure to training and knowledge levels. Second, convenience sampling may have introduced selection bias and limited the representativeness of the sample across all medical colleges in KP. Third, reliance on self‐reported data raises the possibility of social desirability bias and over‐ or underestimation of knowledge and attitudes. Finally, AI is a rapidly evolving field; the tools available and students’ familiarity with them may change quickly, limiting the long‐term generalizability of our findings. However, there are a few limitations that must be considered. The knowledge scale primarily reflects self‐reported familiarity and exposure to AI concepts rather than in‐depth technical competence or clinical application, which may limit construct validity. These findings should be interpreted as exploratory and may not be generalizable to all medical students in Pakistan due to the use of convenience sampling and regional focus.

There is a clear mismatch between enthusiasm for AI‐based learning and students’ technical readiness. Medical schools in KP should urgently consider a staged approach: (1) incorporate core AI literacy into the undergraduate curriculum, (2) run short hands‐on workshops and supervised projects in clinical years, (3) provide faculty development to support teaching and assessment, and (4) include ethical, legal, and patient‐safety‐focused training. Future research should evaluate the effectiveness of these interventions longitudinally and explore objective measures of competence (performance‐based assessments) rather than solely self‐report. Implementation of these recommendations will help ensure that future Pakistani physicians are prepared to use AI responsibly and effectively.

## 5 Conclusion

In our study, most medical students (62.8%) demonstrated a moderate level of knowledge regarding AI‐based learning, whereas 30.4% achieved a high level and 6.9% had low knowledge. Knowledge differed significantly across academic years (*p* = 0.013), with third‐year students attaining the highest scores and first‐year students the lowest. In contrast, attitudes and perceptions were overwhelmingly favorable. A total of 74.2% of students expressed positive attitudes, 25.0% were neutral, and only 0.8% were negative. Similarly, 73.7% of participants had positive perceptions, 25.5% neutral, and 0.8% negative, with no significant differences across academic years. These results reveal a clear mismatch: Although nearly three‐quarters of students hold positive attitudes and perceptions toward AI in medical education, less than one‐third possess high knowledge. This underscores the urgent need for structured AI‐related education in undergraduate medical curricula in KP.

## Author Contributions

Conceptualization: Danyal Khan and Muhammad Dawood. Methodology: Danyal Khan and Muhammad Dawood. Formal analysis: Husna Irfan Thalib and Ihtiram Ullah. Data curation: Ihtiram Ullah and Sadia Yasir Khan. Investigation: all authors. Writing – original draft: Husna Irfan Thalib and Danyal Khan. Writing – review and editing: Farasat Maheen and Sadia Yasir Khan. Supervision: Farasat Maheen. All authors have read and approved the final manuscript.

## Funding

The authors have nothing to report.

## Ethics Statement

The study protocol was reviewed and approved by the Institutional Review Board of Gomal Medical College, Dera Ismail Khan (Approval No. GMC/IRB/2025‐032, dated March 10, 2025).

## Consent

Written informed consent was obtained from all participants prior to their inclusion in the study.

## Conflicts of Interest

The authors declare no conflicts of interest.

## Supporting information




**Supporting File 1**: puh270293‐sup‐0001‐SuppMat.pdf

## Data Availability

The data that support the findings of this study are available on request from the corresponding author. The data are not publicly available due to privacy or ethical restrictions. The corresponding author had full access to all of the data in this study and takes complete responsibility for the integrity of the data and the accuracy of the data analysis.
