# Supplementary material for: Knowledge, Attitude, and Perception of Medical Students Toward AI‐Based Learning: A Cross‐Sectional Study
Source: Public Health Chall. 2026 Jun 6;5(2):e70293. doi: 10.1002/puh2.70293 (PMC13241693; doi:10.1002/puh2.70293)
Supplement: Supplementary file 1 — Supporting File 1: puh270293‐sup‐0001‐SuppMat.pdf [file PUH2-5-e70293-s001.pdf]

SURVEY

"Knowledge, perception and Attitude of Medical Students of KPK Towards AI-Based Learning"

DEMOGRAPHICS

NAME OF INSTITUTE ____________________

YEAR OF STUDY: ○ 1st Year ○ 2nd Year ○ 3rd Year ○ 4th Year ○ Final Year

GENDER: ○ Male ○ Female

RESIDENCE STATUS: ○ Hostel ○ Day-Scholar

CONSENT AND INSTRUCTIONS:

Your participation is voluntary, and you may choose to skip any question or withdraw from the survey at any time without any consequences. All responses will be kept anonymous and will be used solely for research purposes. There are no known risks associated with participation in this study.

SIGNATURE ____________________

KNOWLEDGE QUESTIONS:	Yes	No	Don't Know
Have you ever heard about the use of artificial intelligence in medical education	○	○	○
Do you know AI can be used in clinical practice?	○	○	○
Have you ever heard a talk or read material specifically about AI based learning	○	○	○
Have you been formally taught about AI or its applications in your curriculum?	○	○	○
Do you think AI can help identify knowledge gaps in students?	○	○	○
Do you know the difference between machine learning and deep learning?	○	○	○
Do you know that AI can be used for personalized study pathways?	○	○	○

ATTITUDE QUESTIONS:	RATING SCALE:				
	Strongly Agree	Agree	Neutral	Disagree	Strongly Disagree
All medical students should receive teaching in artificial intelligence					
Teaching in artificial intelligence will be beneficial for my career					
At the end of my medical degree, I will be able to use basic healthcare artificial intelligence tools if necessary.					
At the end of my medical degree, I will have a better understanding of the methods used to assess healthcare artificial intelligence algorithm performance					
Artificial intelligence will improve medicine in general					
These introduction of AI in medical training frighten me					

PERCEPTION QUESTIONS:	RATING SCALE:				
	Strongly Agree	Agree	Neutral	Disagree	Strongly Disagree
I believe AI will play a significant role in the future of medical education.					
AI-based tools can make learning more engaging and effective					
I am confident in my ability to understand and use AI tools for learning.					
I think AI can complement traditional teaching methods					
The use of AI in education may reduce the need for human instructors					
AI can help personalize the learning experience for medical students					
